# Intracolonic Mustard Oil Induces Visceral Pain in Mice by TRPA1-Dependent and -Independent Mechanisms: Role of Tissue Injury and P2X Receptors

**DOI:** 10.3389/fphar.2020.613068

**Published:** 2021-01-21

**Authors:** Rafael Gonzalez-Cano, Ángeles Montilla-García, Gloria Perazzoli, Jesús M. Torres, Francisco J. Cañizares, Eduardo Fernández-Segura, Michael Costigan, José M. Baeyens, Enrique J. Cobos

**Affiliations:** ^1^ Department of Pharmacology, Faculty of Medicine, University of Granada, Granada, Spain; ^2^ Institute of Neuroscience, Biomedical Research Center, University of Granada, Granada, Spain; ^3^ Instituto de Investigación Biosanitaria ibs.GRANADA, Granada, Spain; ^4^ Department of Anatomy and Embryology, Faculty of Medicine, University of Granada, Granada, Spain; ^5^ Department of Biochemistry, Molecular Biology and Immunology, Faculty of Medicine, University of Granada, Granada, Spain; ^6^ Department of Histology, Faculty of Medicine, University of Granada, Granada, Spain; ^7^ Departments of Anesthesia and Neurobiology, Boston Children's Hospital, Harvard Medical School, Boston, MA, United States; ^8^ Teófilo Hernando Institute for Drug Discovery, Madrid, Spain

**Keywords:** mustard oil, visceral pain, resiniferatoxin, TRPA1, TRPV1, P2X

## Abstract

Both TRPA1 and purinergic P2X receptors have been proposed as potential targets for the treatment of visceral pain. We found that the intracolonic administration of a low dose mustard oil (0.5%), a well-known TRPA1 agonist, produced nociceptive responses and abdominal wall referred mechanical hyperalgesia, without inducing apparent tissue damage. Both nociceptive responses and referred hyperalgesia were abolished by the ablation of TRPV1-expressing neurons (and the consequent ablation of TRPA1+ nociceptors) by resiniferatoxin (RTX) treatment, and by the TRPA1 antagonist AP18. However, a higher dose of mustard oil (2.5%) damaged the colonic epithelium and induced pERK activation in the spinal cord, and these processes were clearly independent of TRPV1-expressing neurons ablated by RTX. This higher dose of mustard oil induced nociceptive responses and referred mechanical hyperalgesia which were insensitive or only slightly sensitive to resiniferatoxin or AP18, but were markedly reduced by the P2X antagonist TNP-ATP, which is known to inhibit nociceptive actions induced by ATP released from injured tissues. In conclusion, whereas a low dose of intracolonic mustard oil induces visceral pain in a manner fully dependent on TRPA1 actions, when a high dose of this chemical irritant is used, visceral pain becomes mostly independent of TRPA1 activation but clearly enhanced by ATP purportedly released by the damaged colonic epithelium. Therefore, TRPA1 inhibition is not sufficient to substantially decrease visceral pain during tissue injury, whereas purinergic antagonism appears to be a more effective strategy.

## Introduction

Visceral pain is encountered very frequently in clinical practice, and it is a major reason for seeking medical care (Drewes et al., 2020). The cation channel TRPA1 (transient receptor potential ankyrin 1) has been proposed as a potential target for the treatment of this particular type of pain (e.g., Lapointe and Altier, 2011). Mustard oil (allyl isothiocyanate) is the pungent compound in mustard, horseradish, and wasabi (Bandell et al., 2004). It has been shown that mustard oil, as well as other electrophilic chemicals (such as formalin), can directly activate TRPA1 (Jordt et al., 2004; Macpherson et al., 2007; McNamara et al., 2007), which is located in a subset of TRPV1-expressing nociceptors (Story et al., 2003). When mustard oil is administered in the colon of rodents, it induces nociceptive responses immediately thereafter due to the direct activation of the nociceptors mentioned above, and these responses are followed by mechanical hyperalgesia referred to the abdominal wall (Laird et al., 2001). This latter process is the result of central sensitization (Urban and Gebhart, 1999), which can be evidenced by the phosphorylation (activation) of extracellular signal-regulated kinases (ERK1/2) in the spinal cord (Gao and Ji, 2009). In addition, activation of TRPA1-expressing neurons by mustard oil leads to neurogenic inflammation that promotes plasma extravasation and edema, contributing to pain (Matsuda et al., 2019).

It is worth noting that not all effects of mustard oil are of neuronal origin, as it has been shown that this chemical irritant is able to damage the colonic mucosa even after denervation (Laird et al., 2000). In addition, although injection of low doses of this algogenic chemical into somatic tissue exclusively activates TRPA1, high doses can robustly activate other subsets of nociceptive neurons apart from TRPA1-expressing nociceptors (Braz and Basbaum, 2010). These TRPA1-independent effects of high doses of mustard oil may be attributable to tissue injury and the release of proalgesic mediators by damaged cells, rather than to direct neuronal actions. Consequently, high doses of mustard oil may be useful to study pain induced by tissue injury. ATP acts on neuronal P2X receptors, and it is known to be one of the main algogenic chemicals released during cell damage (Cook and McCleskey, 2002). In fact, purinergic antagonism has been proposed as a promising therapeutic tool for pain relief during inflammatory bowel diseases such as Crohn’s disease and ulcerative colitis (Burnstock, 2017), which are characterized by macroscopic colonic lesions that undoubtedly contribute to pain (Gecse and Vermeire, 2018). However, to our knowledge the contribution of TRPA1 (direct neuronal activation) and P2X receptors (ATP from the injured tissue) to mustard oil-induced visceral pain has never been studied.

Taking into account these antecedents, we aimed to study the contribution of TRPA1 and purinergic receptors to the nociceptive responses and referred hyperalgesia induced by low and high doses of intracolonic mustard oil. First we tested whether treatment with resiniferatoxin, a drug able to selectively ablate TRPV1-expressing neurons (Montilla-García et al., 2018) (which, as described above, contain TRPA1+ nociceptors), alters the behavioral manifestations, histological alterations in the colon (such as the appearance of neurogenic edema and overt tissue damage) and neuronal activation in the spinal cord (measured as ERK phosphorylation) induced by intracolonic mustard oil. Second, we tested the effects of the purinergic antagonist TNP-ATP (Honore et al., 2002) on the behavioral alterations induced by low and high doses of mustard oil, and compared them with those induced by the pharmacological antagonism of TRPA1 by AP18 (Tsubota-Matsunami et al., 2012).

## Methods

### Experimental Animals

The experimental animals were adult female CD-1 mice (Charles River, Barcelona, Spain) weighing 25–30 g. The animals were acclimated in our animal facilities for at least 1 week before testing, and were housed in colony cages (1145T Tecniplast, Varese, Italy) with poplar wood granulate as bedding material (Safe, Augy, France) in temperature- and light-controlled rooms (22 ± 1°C, lights on at 08.00 h and off at 20.00 h, air replaced every 20 min). A standard laboratory diet (Harlan Teklad Research diet, Madison, WI, United States) and tap water were available ad libitum until the beginning of the experiments. Testing took place during the light phase (from 9.00 to 15.00 h) and at random times throughout the estrous cycle. The mice were handled in accordance with international standards (European Communities Council directive 2010/63), and the procedures were approved by the Research Ethics Committee of the University of Granada. To decrease the number of animals in this study, we used the same mice for behavioral studies, histological analysis and immunostaining when possible.

### Drugs and Drug Administration

We evaluated the effects of the selective TRPA1 antagonist AP18 (4-(4-chlorophenyl)-3-methylbut-3-en-2-oxime) and the purinergic antagonist TNP-ATP (2′,3′-O-(2,4,6-trinitrophenyl) adenosine 5′-triphosphate tetrasodium salt) (both supplied by Sigma-Aldrich, Barcelona, Spain) on mustard oil-induced nociceptive behaviors and referred hyperalgesia. AP18 was dissolved in 7.5% DMSO with 0.5% Tween 80, and brought up to 100% volume with PBS. TNP-ATP was dissolved in physiological saline. AP18 (10 mg/kg) or TNP-ATP (25 µg/kg), or their solvents, were administered intraperitoneally in a volume of 10 ml/kg, 30 and 5 min before the intracolonic administration of mustard oil, respectively, as these drugs reportedly showed activity when given via this route at these times before the use of a chemical algogen (Xu et al., 2008; Tsubota-Matsunami et al., 2012). Drug solutions were prepared immediately before the start of the experiments.

### Assessment of Nociceptive Behaviors and Referred Hyperalgesia Induced by Intracolonic Mustard Oil

The dose of mustard oil administered to assess visceral pain in mice varies considerably between studies, and typically ranges from 0.5% to 2.5% (Laird et al., 2001; Li et al., 2013; Sałaga et al., 2015; Zielińska et al., 2015). Therefore, we selected the doses of 0.5 and 2.5% mustard oil as the low and high doses for comparison in most experiments reported here. The mice were housed in individual transparent plastic boxes (7 × 7 × 13 cm) on an elevated platform with a wire mesh floor, and small mirrors behind and below the chambers facilitated observations of the animal’s behaviors. After a 40 min habituation period, animals were removed from the compartments, and after petroleum jelly was applied on the perianal area to avoid stimulation of somatic areas, 50 μL mustard oil (Sigma-Aldrich) solution (from 0.5 to 2.5%) dissolved in PEG 400 (Bourgeois et al., 2014) was instilled into the colon via the anus through a thin, round-tipped cannula (external diameter, 0.61 mm; length, 4 cm) connected to a 1710 TLL Hamilton microsyringe (Teknokroma, Barcelona, Spain). In control animals the same volume of mustard oil vehicle was instilled intracolonically. After instillation, the animals were immediately returned to the compartment, where the numbers of nociceptive behaviors (licking, stretching and contraction of the abdomen) were counted for 20 min, divided into four periods of 5 min each (Laird et al., 2001; González-Cano et al., 2013).

After evaluation of the pain-related behaviors described above, the presence of referred hyperalgesia was determined by testing the number of withdrawal responses to punctate mechanical stimulation of the abdomen. A von Frey filament (Touch-Test Sensory Evaluators, North Coast Medical Inc., Gilroy, CA, United States) calibrated to 0.16 g (3.22 mN) was applied to the lower/mid abdomen, avoiding the perianal and external genitalia areas. The filament was applied 10 times for 2 s each with between-application intervals of at least 5 s, and the number of positive responses out of 10 applications was counted. The response to the filament was considered positive if immediate licking or scratching of the application site, sharp retraction of the abdomen, or jumping were observed.

The experimenter who evaluated the behavioral responses was blinded to the treatment of the experimental animals. Each animal was used only once and received a single concentration of mustard oil or its vehicle and a single dose of one drug or its solvent.

### Evaluation of Nociceptive Responses Induced by the Intraplantar Injection of Formalin or Mustard Oil

Nociceptive responses induced by the intraplantar administration of the chemical irritants were assessed as previously described (Shields et al., 2010), with slight modifications. Briefly, 20 μL of a low dose (0.5%) of diluted formalin (Sigma-Aldrich) dissolved in physiological saline, or mustard oil prepared as described in the preceding section, was injected intraplantarly into the dorsal surface of the right hind paw with a 1710 TLL Hamilton microsyringe (Teknokroma) and a 30 1/2-gauge needle. Immediately after injection the animal was placed in a glass cylinder for observation. In control animals the same volume of the vehicle used for formalin or mustard oil was administered intraplantarly. The time spent licking or biting the injected paw was recorded for 10 min immediately after the injection.

### *In Vivo* Ablation of TRPV1-Expressing Neurons

We used resiniferatoxin (RTX, Tocris Cookson Ltd., Bristol, United Kingdom) as a “molecular scalpel” to selectively ablate TRPV1-expressing neurons. After anesthesia was induced with isoflurane (IsoVet®, B. Braun, Barcelona, Spain), each animal received a single dose of RTX (50 µg/kg) dissolved in 10% Tween 80 and 10% ethanol in physiological saline (0.9% NaCl) via intraperitoneal injection (Montilla-García et al., 2017). Animals in the control group received an equal volume of vehicle. After the intraperitoneal injection, the mice were housed in their home cages for the next 5 days, after which behavioral testing or sample collection took place.

### Determination of *TrpA1* and *TrpV1* Expression

RNA samples were extracted from 12 L6 dorsal root ganglia (DRG) pooled from six different animals. Total RNA was isolated on phenol/chloroform medium using Trizol reagent (Invitrogen, Thermo Fisher Scientific, Waltham, MA, United States) and ethanol precipitation, according to the instructions of the Sanger Institute^©^ (ftp://ftp.sanger.ac.uk/pub/resources/mouse/sigtr/RTPCR.pdf). RNA concentration and quality were assessed spectrophotometrically with a NanoDrop ND-1000 apparatus, and electrophoretically with ethidium bromide staining.

First-strand cDNA was synthesized using 1 µg of each RNA sample in a reaction volume of 20 µL containing 5 mM MgCl_2_, 1× RT buffer, 1 mM dNTP, 1 U/µL RNase inhibitor, 2.5 µM Oligo (dT)16, and 2.5 U/µL MuLV reverse transcriptase (Applied Biosystems, Madrid, Spain). The mix was incubated at 42°C for 15 min, and at 99°C for 5 min.

Relative quantification of *TrpA1* and *TrpV1* mRNA levels in mouse DRGs was carried out with real-time PCR. Primers that are spanned by introns were designed to amplify *TrpA1* and *TrpV1*, and murine β-actin was used to normalize the total amounts of mRNA. Primer sequences, PCR product sizes and GenBank accession numbers for each gene are given in Table 1. PCR reactions were set up by combining diluted cDNA (5 μL) as the template with a 0.5 μM final concentration of each primer pair and 12.5 μL of 2× SYBR Green PCR Master Mix (Promega, Madrid, Spain), and were adjusted to a final volume of 25 μL with sterile, nuclease-free water (Bio-Rad, Hercules, CA, United States). Samples were initially denatured at 94°C for 2 min, followed by 40 cycles of 30 s for denaturation at 94°C, 30 s of annealing at 60°C, and 30 s for extension at 72°C on a Techne Quantica™ Real-time PCR system. Melting curve analysis was used to verify amplification specificity. All reactions were run in triplicate with non-template controls. Three independent biological replicates were used under each condition.

**TABLE 1:** Primer sequences (5′-3′) and PCR product sizes.

GenBank Acc. No	Primer		Forward	Reverse	Product size (bp)
*NM_177781*	Trpa1	ACA​AGA​AGT​ACC​AAA​CAT​TGA​CAC​A		TTA​ACT​GCG​TTT​AAG​ACA​AAA​TTC​C	243
*NM_001001445*	Trpv1	CAT​CAT​CAA​CGA​GGA​CCC​AG		AAC​CAG​GGC​AAA​GTT​CTT​CC	109
*NM_007393*	β-actin	TGT​TAC​CAA​CTG​GGA​CGA​CA		GGG​GTG​TTG​AAG​GTC​TCA​AA	165

### Histology

After deep anesthesia was induced with isoflurane, the mice were perfused intracardially with 20 ml saline followed by 4% paraformaldehyde solution (Scharlab, Barcelona, Spain). The colon was removed and post-fixed in 4% paraformaldehyde for 2 h at 4°C. Samples were dehydrated and embedded in paraffin blocks. Slices were deparaffinized with xylene and rehydrated before tissue sections (5 µm) were stained with hematoxylin-eosin (Panreac, Castellar del Vallès, Spain). Morphometric analysis of each stained section was done with ImageJ software (version 1.48, Wayne Rasband, NIH, Bethesda, MD, United States), and the area of the submucosa (located between the lamina propria and muscularis) was measured and normalized with respect to the total area of the colon section, as an index of edema. Images were acquired with a Nikon Eclipse 50i microscope equipped with a DS-Ri1 camera (Nikon Instruments Europe BV, Amsterdam, Netherlands) at magnifications of 4× and 10×.

### Immunohistochemistry

Mice were perfused transcardially as described above, and the L6 DRG and corresponding L3 spinal cord segment were collected. The tissues were post-fixed, dehydrated and embedded in paraffin as described above before immunostaining.

TRPV1 and NeuN immunostaining was done in the L6 DRG, where the majority of colorectal afferents are located (Robinson et al., 2004; Guo et al., 2019). After deparaffinization, tissue sections were incubated with blocking solution (5% normal donkey serum, 0.3% Triton X-100 in TBS) for 1 h at room temperature. The sections were then incubated for 1 h at room temperature with a goat anti-TRPV1 antibody (sc-12498, 1:100, Santa Cruz Biotechnology, Heidelberg, Germany) in blocking solution. After incubation, the sections were washed three times for 10 min each, and incubated for 1 h with the secondary donkey anti-goat Alexa Fluor-488 antibody (A-11055, 1:500; Invitrogen) and a conjugated mouse anti-NeuN antibody (MAB377A5, 1:500, Merck Millipore, Madrid, Spain). The sections were then washed three times for 10 min each, and mounted with ProLong® Gold Antifade Mountant (Life Technologies, Alcobendas, Spain). Images were acquired with a confocal laser-scanning microscope (Model A1, Nikon Instruments Europe BV).

Tissue sections from the L3 spinal cord segment were treated for antigen retrieval by steam heating in 0.01 M citrate buffer, pH 6.0, during 20 min. Endogenous peroxidase activity was quenched with 3% H_2_O_2_ in methanol, pH 7.4, for 15 min. The sections were then washed in TBS with 0.1% Tween 20, and incubated in blocking solution (2.5% rabbit serum) for 20 min. This was followed by incubation with a primary antibody against phospho-extracellular signal-regulated kinases (pERK1/2) (4370S, 1:200; Cell Signaling Technology, Danvers, MA, United States) in an incubation chamber at room temperature for 60 min. An ImmPRESS^®^ HRP Anti-Rabbit IgG (peroxidase) Polymer Detection Kit (MP-7401, Vector Laboratories, Burlingame, CA, United States) was used according to the manufacturer’s instructions, and the immunoreaction was visualized with an ImmPACT™ DAB peroxidase (HRP) substrate (SK-4105, Vector Laboratories) for 20 s.

### Statistical Analysis

When several means were compared, one-way or two-way analysis of variance (ANOVA) was used depending on the experiment, followed by the Bonferroni post-hoc test. When two means were compared, an unpaired Student’s *t* test was used. Real time qPCR data were analyzed with Student’s *t* test statistics and the comparative Ct method (2-∆∆Ct method) (Livak and Schmittgen, 2001). Raw Ct data were normalized against β-actin expression. In all comparisons, the differences between values were considered significant when the *p* value was below 0.05. All statistical analyses was done with the SigmaPlot 12.0 program (Systat Software Inc., San Jose, CA).

## Results

### Nociceptive Responses and Referred Hyperalgesia Induced by Different Doses of Intracolonic Mustard Oil

**FIGURE 1:**
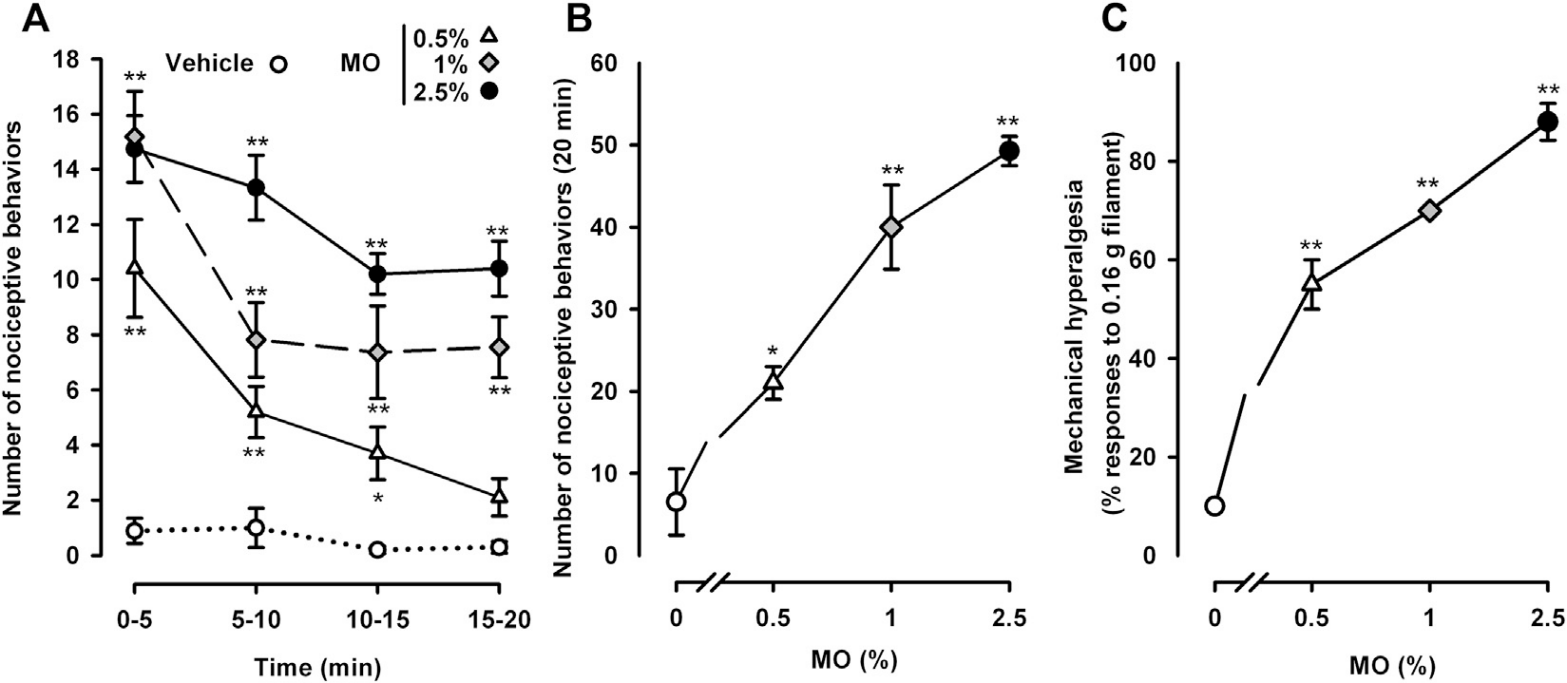
Nociceptive behaviors and referred hyperalgesia induced by different doses of intracolonic mustard oil. **(A)** Time-course of nociceptive behaviors in mice during 20 min after the intracolonic administration of mustard oil (MO, 0.5, 1.0 or 2.5%) or its vehicle, measured in 5 min periods. **(B)** Nociceptive behaviors across the entire observation period (0–20 min) after the administration of MO or its vehicle in mice from the experimental groups shown in **(A)**. **(C)** Responses to mechanical stimulation with a 0.16 g von Frey filament in the abdominal wall of mice 20 min after instillation of MO or its vehicle. **(A–C)** Each point and vertical line represents the mean ± SEM of values obtained in 8 -10 mice per group. Statistically significant differences between the values in mice treated with MO or its vehicle: *p* < 0.05, *p* < 0.01; two-way **(A)** or one-way **(B** and **C)** ANOVA followed by Bonferroni test.

Animals treated intracolonically with the mustard oil vehicle showed almost no measurable pain-related responses (e.g., licking, stretching, or contraction of the abdomen) during the whole observation period (20 min) (Figure 1A). The intracolonic administration of mustard oil at any of the three doses tested (0.5, 1.0 or 2.5%) induced pain-related behaviors which were maximal in the period immediately after administration (0–5 min) (Figure 1A). Although the number of nociceptive behaviors induced by each of the three doses did not differ markedly during the first 5 min after application, the duration of the nociceptive response was dose-dependent. When the 0.5% concentration of the chemical irritant was used, behavioral responses decreased rapidly after 5 min, and disappeared almost completely after 15–20 min. However, when the 2.5% concentration of mustard oil was used, nociceptive responses were sustained with only a slight decrease during the 20 min observation period. The 1.0% dose of mustard oil showed an intermediate pattern: nociceptive behaviors decreased during the first 5–10 min and then remained stable during the remaining 10 min (Figure 1A).

The dose dependence of the behavioral effects induced by mustard oil became more obvious when we summed the total number of pain-like responses throughout the 20 min evaluation period after the chemical irritant was applied (Figure 1B).

We also assessed abdominal wall referred hyperalgesia to punctate mechanical stimulation after mustard oil instillation. Animals treated with the chemical algogen vehicle responded only 10% of the times when stimulated with a thin (0.16 g) von Frey filament (Figure 1C). After the instillation of 0.5% mustard oil, the animals showed exacerbated responses in the von Frey test, responding to 55 ± 5% of the stimulations (Figure 1C). Sensitization to mechanical stimulation was even more evident in tests with 1.0 or 2.5% mustard oil, showing a clear dose dependence with responses to as many as 88 ± 3.74% of the stimulations in the group that received the 2.5% concentration of mustard oil (Figure 1C).

We selected the 0.5 and 2.5% doses of mustard oil as the low and high dose, respectively, for subsequent experiments.

### Differential Effects of the Ablation of TRPV1-Expressing Neurons on Nociceptive Behaviors and Referred Hyperalgesia Induced by Low and High Doses of Mustard Oil

**FIGURE 2:**
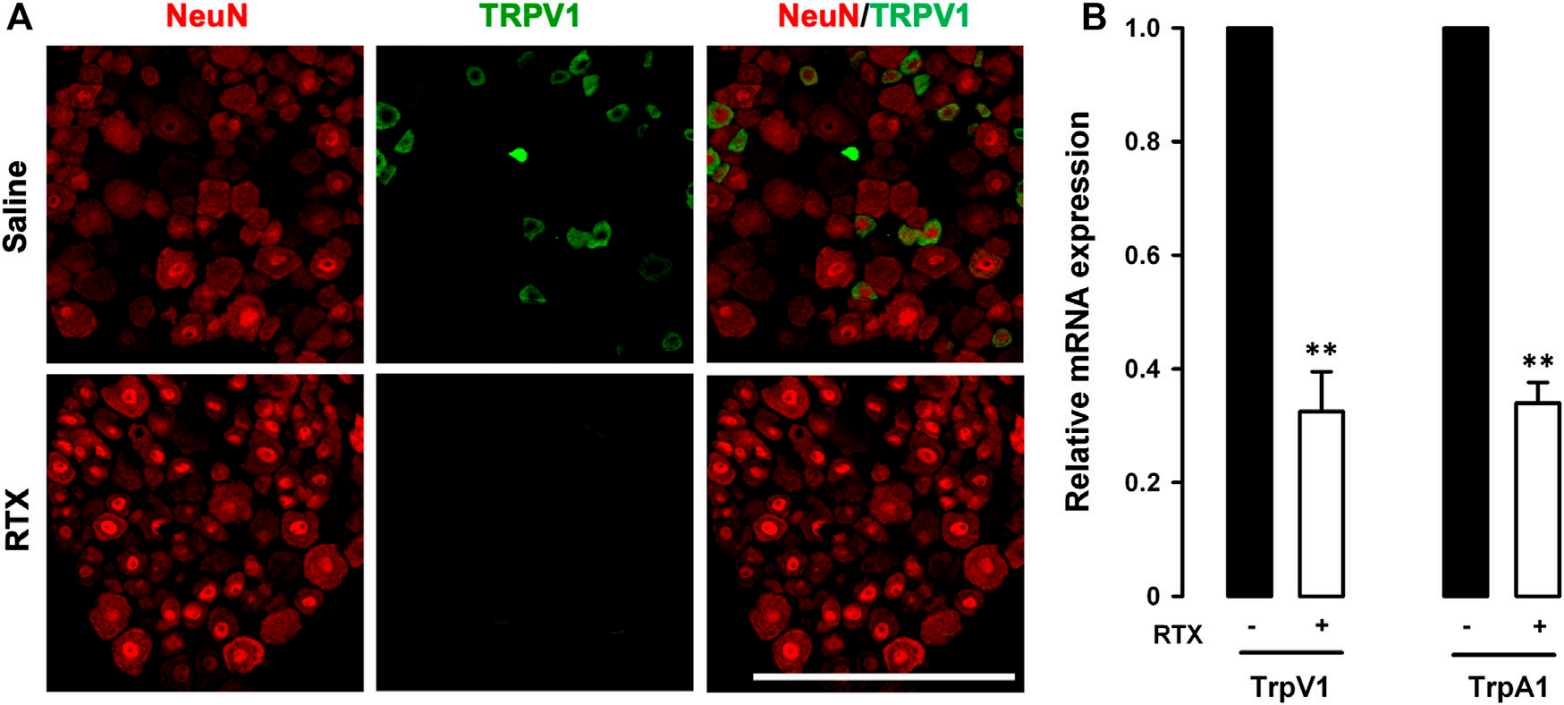
Resiniferatoxin ablates the majority of TRPV1-expressing neurons. **(A)** Double-labeling of NeuN (red) and TRPV1 (green) in the L6 dorsal root ganglion (DRG). Top panels: samples from control (saline-treated) mice. Bottom panels: samples from mice treated with resiniferatoxin (RTX). Scale bar, 100 mm. **(B)** Relative quantification of *TrpA1* and *TrpV1* mRNA levels with real-time PCR in L6 DRGs from mice treated with RTX or saline. Statistically significant differences between the values in mice treated RTX or saline: **p < 0.01 (two-way ANOVA followed by Bonferroni test).

We first performed double immunostaining in the L6 DRG to label the pan-neuronal marker NeuN and TRPV1 in tissue samples from mice treated with RTX or its solvent. While NeuN was found to be expressed in the somas of all DRG neurons, TRPV1 was only labeled in some small neurons in control mice treated with the RTX solvent (Figure 2A, upper panels). In animals treated with RTX we detected no appreciable TRPV1 staining, although NeuN labeling was still present (Figure 2A, lower panels), suggesting that neuronal ablation was restricted to the TRPV1+ population. Identical results were found in the L4 DRG (data not shown). As expected, and as a result of the ablation of TRPV1-expressing neurons, mRNA for *TrpV1* in the L6 DRG was clearly reduced (Figure 2B). Furthermore, mRNA for *TrpA1* was also markedly reduced in RTX-treated animals, to the same extent as *TrpV1* transcripts (Figure 2B). These results suggest that TRPA1+ neurons are a part of the TRPV1-expressing neuronal population.

It has been reported that the nociceptive effects of low doses of formalin and mustard oil injected intraplantarly were abolished in TRPA1 knockout mice (Braz and Basbaum, 2010). Therefore, to test whether RTX-induced ablation of TRPV1-expressing neurons led to the functional suppression of TRPA1 activity, we evaluated nociceptive responses induced by the intraplantar injection of a low dose (0.5%) of formalin or mustard oil in animals treated with solvent or RTX. Formalin and mustard oil both induced robust licking/biting of the injected hind paw in control animals, whereas the responses were markedly reduced in RTX-treated mice (Supplementary Figures S1A,B). Therefore, in our experimental conditions, RTX treatment induced significant ablation of TRPV1-expressing neurons and a concomitant reduction in TrpA1 transcription, which were accompanied by a decrease in pain responses induced by known TRPA1 activators injected in somatic tissue.

**FIGURE 3:**
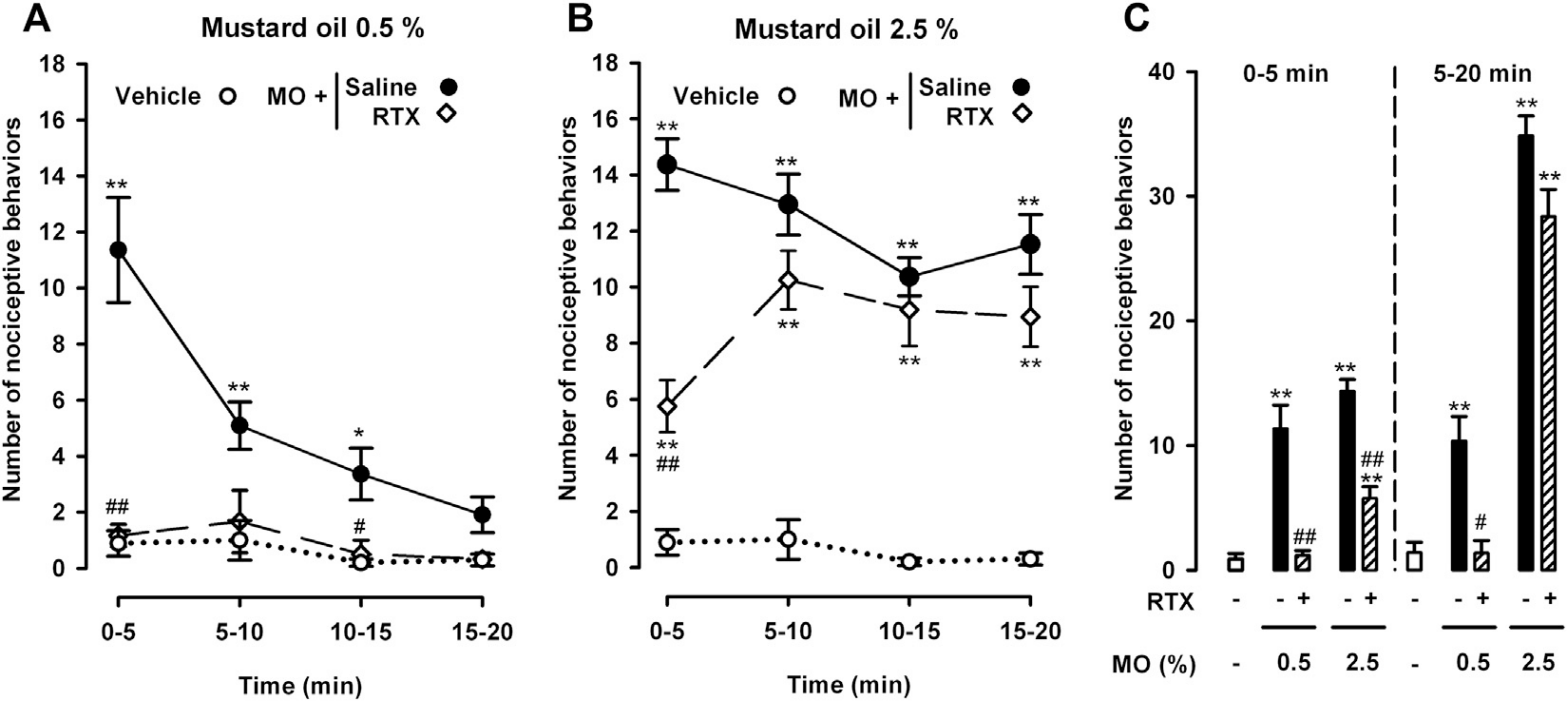
Differential effects of the ablation of TRPV1-expressing neurons on nociceptive behaviors induced by the intracolonic administration of a low and a high dose of mustard oil. Effect of treatment with resiniferatoxin (RTX) or its solvent (saline) on the time-course of nociceptive behaviors in mice during 20 min (measured in 5 min periods) after the intracolonic administration of mustard oil (MO) 0.5% **(A)** and 2.5% **(B)**, or its vehicle **(C)** Nociceptive behaviors in the first 5 min (0–5) and in the following 15 min (5–20) after the administration of MO or its vehicle in mice from the experimental groups shown in **(A)** and **(B)**. **(A–C)** Each point or bar and vertical line represents the mean ± SEM of values obtained in 8–10 mice per group. Statistically significant differences between the values in mice treated with MO or its vehicle: *p < 0.05, **p < 0.01; and between the values obtained in mice given MO and saline or RTX: #*p* < 0.05, ##*p* < 0.01 (two-way ANOVA followed by Bonferroni test).

We then investigated the effect of RTX-induced ablation of TRPV1-expressing neurons on both nociceptive behaviors and abdominal wall referred hyperalgesia induced by a low (0.5%) and a high dose (2.5%) of intracolonic mustard oil. RTX treatment almost fully abolished nociceptive behaviors induced by the low dose of intracolonic mustard oil, during both the peak in pain responses during the first 5 min after mustard oil administration and the subsequent 5 min intervals throughout the 20 min evaluation period (Figure 3A). When we tested the high dose of mustard oil (2.5%) we found a significant albeit partial reduction in the initial nociceptive responses during the 5 min period immediately after administration of the chemical irritant, but after this initial decrease, pain-like responses in RTX-treated animals increased up to values close to those seen in animals treated with the RTX solvent during all subsequent 5 min periods throughout the 20 min evaluation period (Figure 3B).

To facilitate comparisons of the effect of TRPV1+ neuron ablation on the nociceptive responses induced by both doses of intracolonic mustard oil in the initial period after administration of the chemical irritant and at longer time-points, we grouped pain-like responses during the 5–20 min period after each dose of mustard oil separately from the initial recording during the first 0–5 min. This analysis clearly showed full inhibition by RTX of the nociceptive responses induced by 0.5% mustard oil in both the immediate and longer periods after administration of the chemical irritant, and that when 2.5% mustard oil was used, RTX only partially reversed nociceptive responses during the initial 5-min period and was devoid of effect during the remaining 5–20 min (Figure 3C).

**FIGURE 4:**
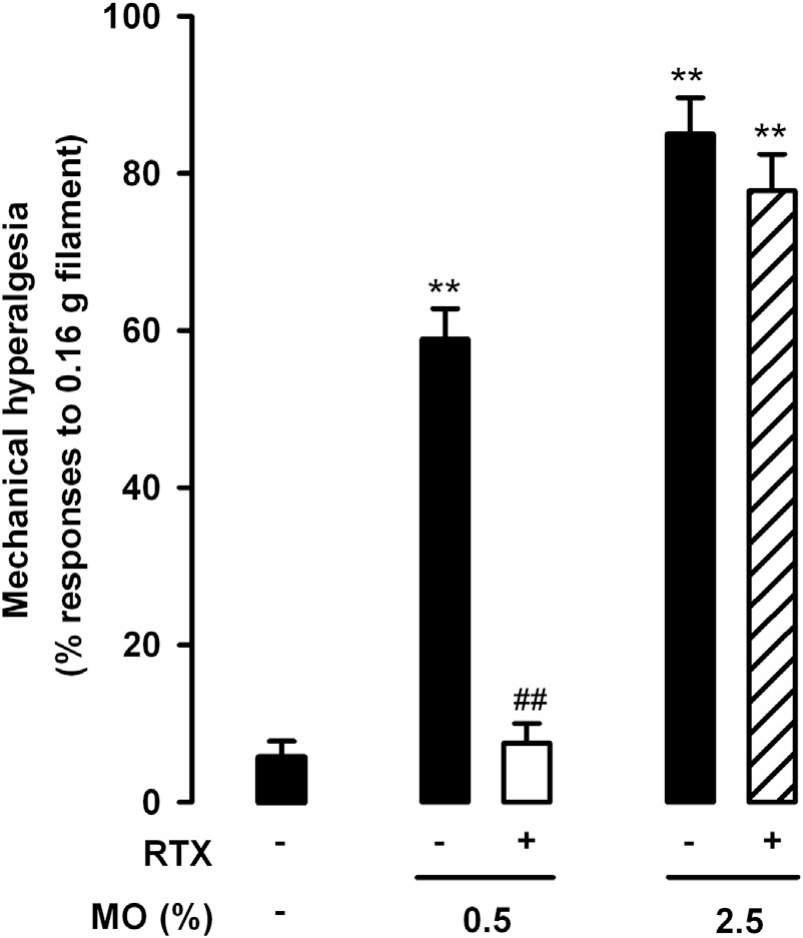
Differential effects of the ablation of TRPV1-expressing neurons on referred hyperalgesia induced by the intracolonic administration of a low and a high dose of mustard oil. Responses to mechanical stimulation with a 0.16-g von Frey filament in the abdominal wall of mice treated with resiniferatoxin (RTX) or its solvent (saline), 20 min after the instillation of MO (0.5 and 2.5%) or its vehicle. Each bar and vertical line represents the mean ± SEM of values obtained in 8–10 mice per group. Statistically significant differences between the values in mice treated with MO or its vehicle: **p < 0.01; and between the values obtained in mice given MO and saline or RTX: ##*p* < 0.01 (two-way ANOVA followed by Bonferroni test).

When we tested abdominal wall referred mechanical hyperalgesia induced by the low and high doses of mustard oil in animals treated with RTX or its solvent, we found that TRPV1 neuron ablation resulted in full reversion of tactile hypersensitivity in the 0.5% mustard oil group, but had no effect in the 2.5% dose group (Figure 4).

Therefore, TRPV1+ neurons (which express TRPA1) appear to be necessary for both the pain-like behaviors and referred hyperalgesia induced by intracolonic 0.5% mustard oil, whereas their role appears to be limited in the nociceptive responses induced by 2.5% mustard oil, and are apparently not involved in tactile hypersensitivity induced by this high dose of the chemical irritant.

### Role of TRPV1-Expressing Neurons in Histological Alterations in the Colon After the Administration of Low and High Doses of Mustard Oil

To determine whether mustard oil induced observable alterations in the colon that could account for the TRPV1 neuron-independent pain responses described in the preceding section, we carried out histological studies in animals treated with RTX or its solvent.

**FIGURE 5:**
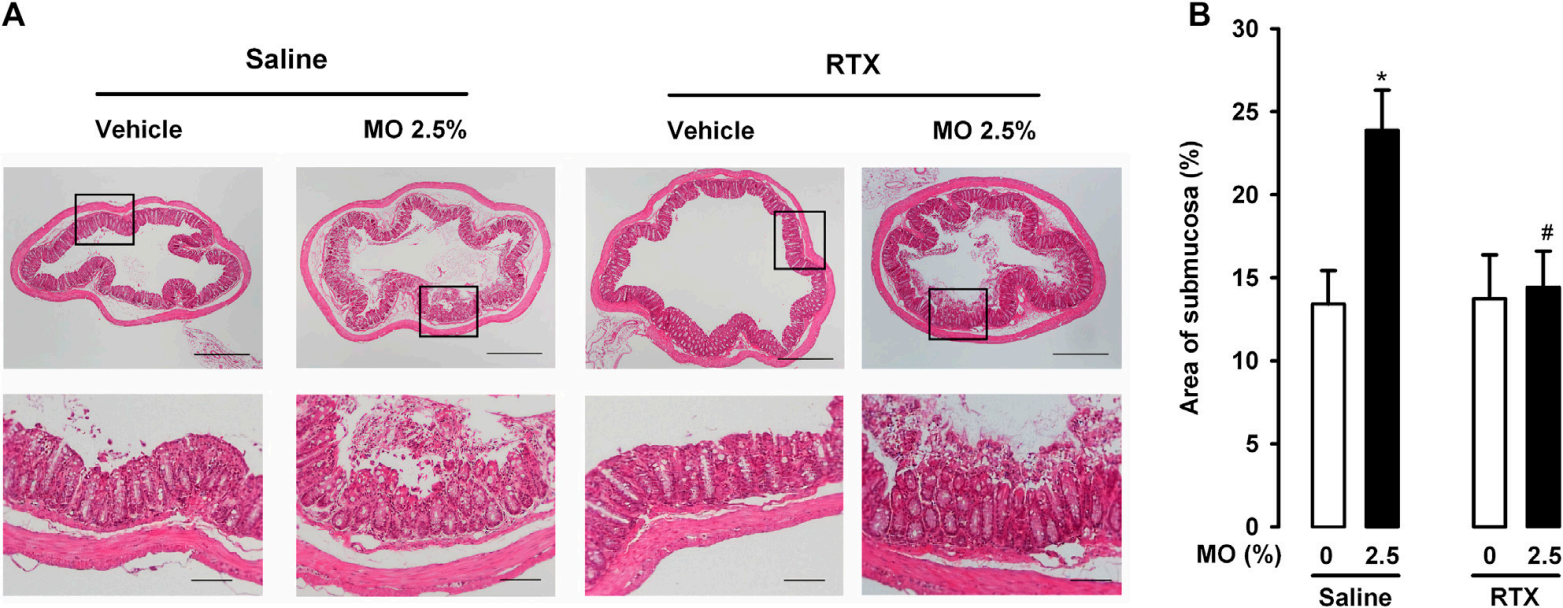
TRPV1-expressing neurons influence edema but not tissue damage after intracolonic administration of a high dose of mustard oil. **(A)** Top: photomicrographs of representative hematoxylin–eosin-stained colon sections in mice given mustard oil (MO) 2.5% or its vehicle, and treated with resiniferatoxin (RTX) or its solvent (saline). Scale bar 250 μm. Bottom: details of the epithelium in the boxed areas in the top panels. Scale bar 100 μm. **(B)** Quantification of submucosa relativized to total area of the colon section (as a measure of edema) in mice given MO or its vehicle and treated with RTX or saline. Each bar and vertical line represents the mean ± SEM of values obtained in four to six mice per group. Statistically significant differences between the values in mice treated with MO or its vehicle: *p < 0.05; and between the values obtained in mice given MO and saline or RTX: #*p* < 0.05 (two-way ANOVA followed by Bonferroni test).

The intracolonic administration of 0.5% mustard oil induced no overt histological alterations, and we observed only mild (nonsignificant) edema in comparison to samples from vehicle-treated mice, measured as the increase in the area of the submucosa relative to the total area of the colon section. The percentage area of submucosa was 13.41 ± 2.01% in animals treated with the vehicle, and 18.14 ± 4.52% in animals treated with the low dose of mustard oil (*p* > 0.05). However, 2.5% mustard oil induced prominent histological alterations, including marked edema (Figure 5A, upper left panels; Figure 5B), as well as severe tissue damage evidenced by loss of epithelium and damage to the crypts (Figure 5A, lower left panels). RTX treatment did not induce obvious changes in the appearance of colon sections from mice treated with the mustard oil vehicle (Figure 5A, upper right panels), but fully attenuated the edema induced by 2.5% of this chemical irritant (Figure 5A, upper right panels; Figure 5B). Interestingly, damage to the epithelium and crypts was still present in animals treated with 2.5% mustard oil despite TRPV1 ablation by RTX (Figure 5A, lower right panels).

Therefore, our findings suggest that although edema induced by the intracolonic administration of a high dose of mustard oil was fully dependent on the presence of TRPV1-expressing neurons, damage to the colonic epithelium appeared to be mediated by mechanisms independent of this neuronal population.

### Contribution of TRPV1-Expressing Neurons to Neuronal Activation (pERK) in the Spinal Cord After the Administration of a High Dose of Mustard Oil

We tested whether stimulation of the colon with 2.5% mustard oil induced the activation (phosphorylation) of ERK1/2 in the L3 spinal cord (innervated by L6 DRG neurons) as a surrogate measure of nociceptive neuron activation, and the dependence of this process on RTX-sensitive neurons. Because both superficial laminae of the spinal cord dorsal horn and lamina X neurons have been implicated in visceral pain (Traub and Murphy, 2002), we carried out these studies in both areas.

**FIGURE 6:**
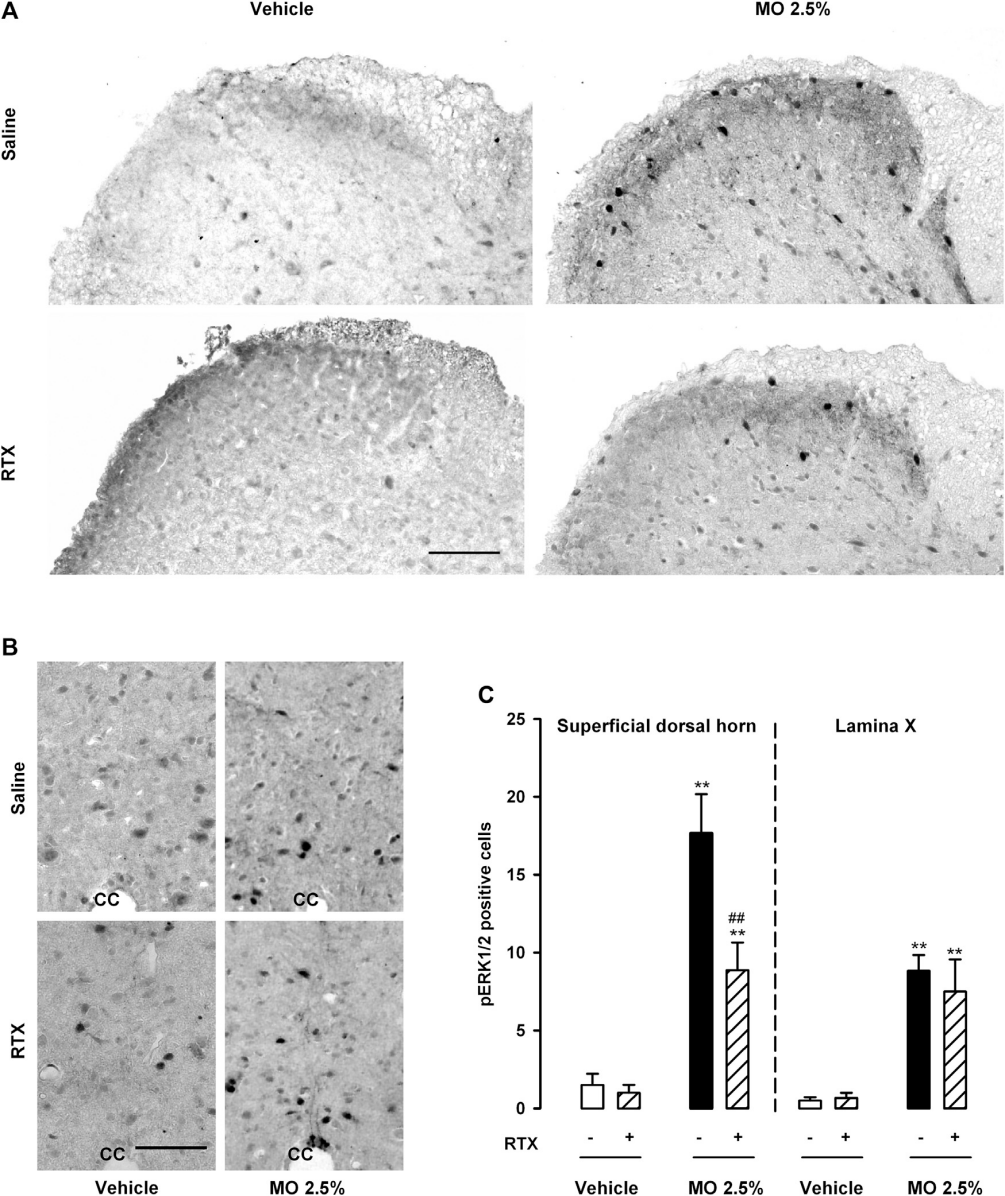
Expression of pERK1/2 in the spinal cord after the intracolonic administration of a high dose of mustard oil is only partly dependent on TRPV1-expressing neurons. **(A)** Representative photomicrographs of pERK1/2 immunostaining in the superficial dorsal horn and **(B)** in the area surrounding the central canal (lamina X) from the L3 spinal cord after the intracolonic administration of 2.5% mustard oil (MO) or its vehicle, in samples from control (saline-treated) animals (top panels) and mice treated with resiniferatoxin (RTX, lower panels). Scale bar 100 μm. **(C)** Quantification of the number of pERK1/2-expressing cells in the superficial dorsal horn and lamina X after the intracolonic administration of 2.5% MO or its vehicle, in samples from mice treated with RTX or saline. Each bar and vertical line represents the mean ± SEM of values obtained in eight animals. Statistically significant differences between the values in mice treated with MO or its vehicle: **p < 0.01; and between the values obtained in the superficial dorsal horn in mice given MO and saline or RTX: ##*p* < 0.01 (two-way ANOVA followed by Bonferroni test).

We found no appreciable pERK1/2 staining in the spinal cord of saline- or RTX-treated mice (in the absence of mustard oil), in the superficial dorsal horn, or the area surrounding the central canal (lamina X). Figures 6A,B (left panels) show representative images, and Figure 6C shows quantitative findings for the numbers of pERK1/2 + cells. In contrast, a significant increase in the number of pERK1/2 + cells was noted in these areas in samples from mice given 2.5% mustard oil intracolonically (Figures 6A–C). RTX treatment markedly reduced pERK1/2 staining in samples from mice treated with 2.5% mustard oil, but only in the superficial dorsal horn, with no effect on pERK1/2 labeling in lamina X, where labeling remained elevated despite the ablation of TRPV1-expressing neurons (Figures 6A–C).

Therefore, 2.5% mustard oil produced marked activation of ERK1/2 in areas of the spinal cord relevant to visceral pain. TRPV1-expressing neurons appeared to be involved in this activation only in the superficial dorsal horn, whereas pERK1/2 activation in lamina X was independent of this neuronal population.

### Effects of Pharmacological Antagonism of TRPA1 and P2X Purinoreceptors on Nociceptive Responses and Referred Hyperalgesia Induced by Low and High Doses of Mustard Oil

Given that 2.5% mustard oil (but not the low 0.5% dose) induced substantial damage to the colonic epithelium, we hypothesized that the TRPV1 neuron-independent behavioral responses we observed with the high dose of mustard oil might be due to substances released during tissue damage. Because ATP is one of the canonical chemical algogens released to the extracellular environment after cell death (Cervero et al., 1993), we studied the effects of the P2X antagonist TNP-ATP on nociceptive behaviors and referred hyperalgesia in animals treated with 0.5 and 2.5% mustard oil, and compared its effects with those of the antagonism of TRPA1 receptors by AP18.

**FIGURE 7:**
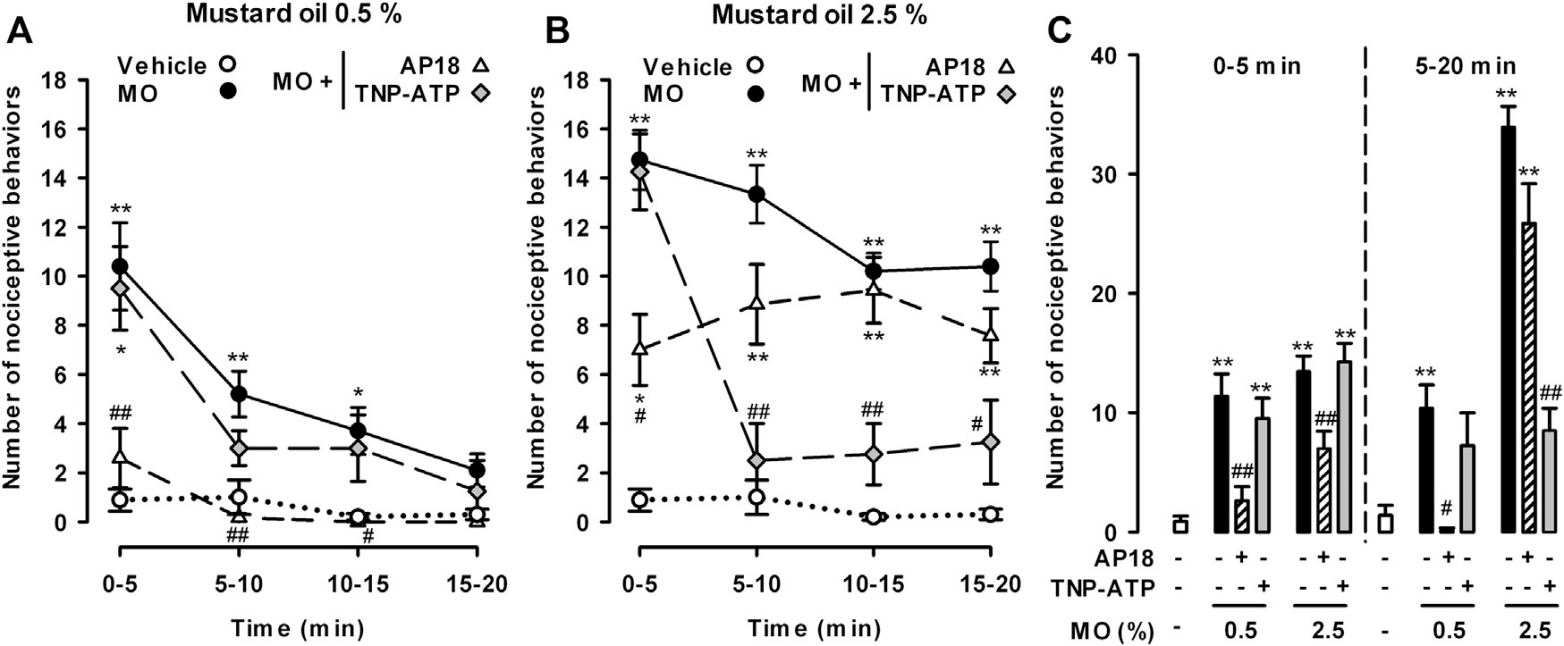
Differential effects of TRPA1 and P2X antagonism on nociceptive behaviors induced by the intracolonic administration of a low and a high dose of mustard oil. Effect of the intraperitoneal administration of AP18 (10 mg/kg) or TNP-ATP (25 μg/kg) on the time-course of nociceptive behaviors in mice during 20 min (measured in 5-min periods) after the intracolonic administration of mustard oil (MO) 0.5% **(A)** and 2.5% **(B)**, or its vehicle **(C)** Nociceptive behaviors in the first 5 min (0–5) and in the subsequent 15 min (5–20) after the administration of MO or its vehicle in mice from the experimental groups showed in **(A)** and **(B)**. **(A–C)** Each point or bar and vertical line represents the mean ± SEM of values obtained in six to eight mice per group. Statistically significant differences between the values in mice treated with MO or its vehicle: *p < 0.05, **p < 0.01; and between the values obtained in mice given MO alone or with AP18 or TNP-ATP: #*p* < 0.05, ##*p* < 0.01 (two-way ANOVA followed by Bonferroni test).

The administration of AP18 or TNP-ATP solvents did not significantly influence pain behaviors induced by the intracolonic administration of either the low or the high dose of mustard oil (data not shown). AP18 fully abolished nociceptive behaviors induced by the low dose of intracolonic mustard oil throughout the 20 min observation period (Figure 7A). However, this TRPA1 antagonist induced only a partial reduction in the initial nociceptive responses during the 5 min period immediately after administration of the high dose of mustard oil, without affecting nociceptive responses during the subsequent 5 min periods up to the end of the 20 min period (Figure 7B). The effects of TNP-ATP on visceral pain induced by mustard oil showed a pattern different to those exerted by AP18. The P2X antagonist did not modify the nociceptive responses induced by 0.5% mustard oil at any time during the observation period (Figure 7A). TNP-ATP was also unable to modify the initial pain responses during the 5 min immediately after 2.5% mustard oil was given, but almost entirely abolished pain responses at longer time-points from 5 to 20 min after the administration of the chemical irritant (Figure 7B).

To facilitate comparisons of the effect of AP18 and TNP-ATP on the nociceptive responses induced by each dose of mustard oil during the initial period after the administration of the chemical irritant and at longer time-points, we grouped the pain-like responses during 5–20 min after the application of each dose of mustard oil separately from the initial responses during the first 0–5 min (Figure 7C). This analysis clearly showed that TRPA1 antagonism generally decreased the nociceptive responses induced by 0.5% mustard oil, whereas when the high dose of the chemical irritant was used, additional mechanisms appeared to be involved in nociceptive responses, particularly 5 min after mustard oil administration and thereafter. Taking into account the marked inhibitory effects of TNP-ATP on late nociceptive responses induced by 2.5% mustard oil, the mechanisms underlying these effects appear to involve the participation of P2X receptors.

**FIGURE 8:**
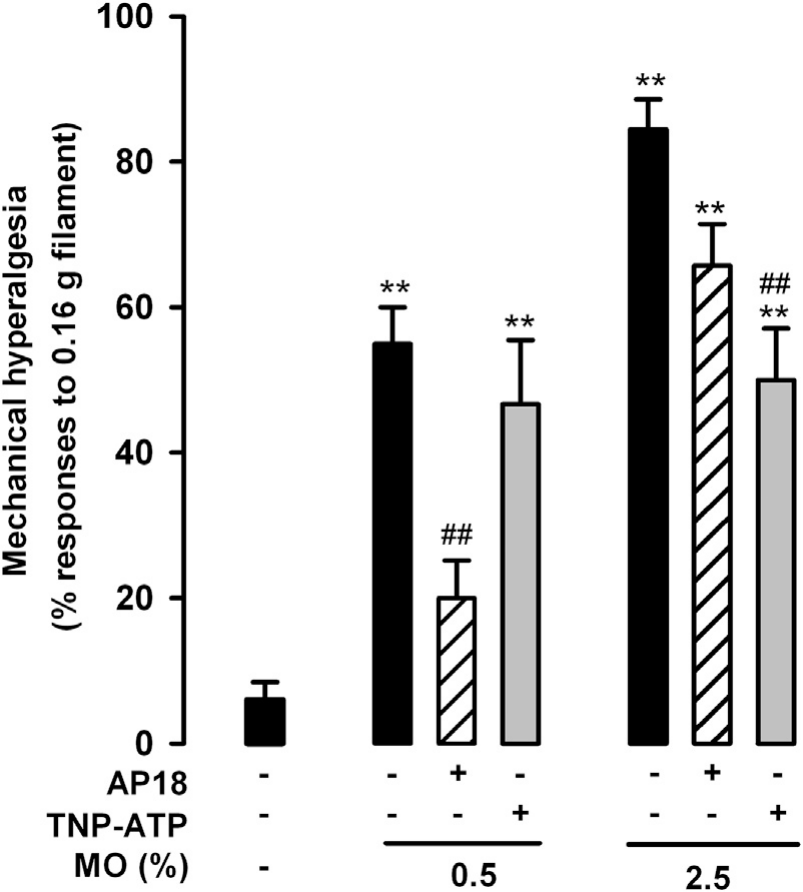
Differential effects of TRPA1 and P2X antagonism on referred hyperalgesia induced by the intracolonic administration of a low and a high dose of mustard oil. Responses to mechanical stimulation with a 0.16-g von Frey filament in the abdominal wall of mice treated intraperitoneally with AP18 (10 mg/kg) or TNP-ATP (25 μg/kg), 20 min after the instillation of MO (0.5 and 2.5%) or its vehicle. Each bar and vertical line represents the mean ± SEM of values obtained in six to eight mice per group. Statistically significant differences between the values in mice treated with MO or its vehicle: **p < 0.01; and between the values obtained in mice given MO alone or with AP18 or TNP-ATP: ##*p* < 0.01 (two-way ANOVA followed by Bonferroni test).

We also tested referred mechanical hyperalgesia induced by the low and high dose of mustard oil in animals treated with AP18 and TNP-ATP. We found that AP18 markedly reversed tactile hypersensitivity when 0.5% mustard oil was used, but showed minimal (nonsignificant) effects in animals given the 2.5% dose of the chemical irritant (Figure 8). On the other hand, TNP-ATP was unable to reverse referred hyperalgesia produced by 0.5% mustard oil although it led to a significant decrease in the tactile hypersensitivity induced by 2.5% of this chemical irritant. These results point again to different roles of TRPA1 and P2X receptors in the pain induced by the different doses of mustard oil tested here.

## Discussion

In this study we show that both nociceptive responses and referred mechanical hyperalgesia in the abdominal wall induced by the intracolonic administration of a low dose of mustard oil (0.5%) were reversed by treatment with the “molecular scalpel” RTX and by the TRPA1 antagonist AP18, but not by the purinergic antagonist TNP-ATP. However, when a higher dose (2.5%) of mustard oil was used, nociceptive responses and referred hyperalgesia were almost entirely unaffected by RTX or AP18, but were substantially inhibited by TNP-ATP.

Our results show that systemic RTX treatment fully abolished TRPV1 staining in the DRG and markedly reduced *TrpV1* mRNA, indicating that RTX successfully ablated the vast majority of TRPV1-expressing neurons. We also show that RTX treatment markedly reduced *TrpA1* mRNA, to the same extent as *TrpV1* mRNA. These results suggest that TRPA1+ neurons were also ablated by this neurotoxin, and are in agreement with the previously described location of TRPA1+ neurons in a subset of TRPV1-expressing nociceptors (Story et al., 2003; La et al., 2011; Le Pichon and Chesler, 2014). Ablation of TRPV1-expressing neurons (and the consequent ablation of TRPA1+ neurons) resulted in the abolishment of nociceptive behaviors induced by the intraplantar administration of a low dose of either formalin or mustard oil, as shown here and in previous studies (Shields et al., 2010), which indicates the functional impairment of TRPA1+ neurons by RTX treatment. Nociceptive behaviors and referred hyperalgesia induced by 0.5% mustard oil given intracolonically were also abolished not only by the ablation of TRPV1-expressing neurons but also by the TRPA1 antagonist AP18, supporting the selective TRPA1-mediated actions on visceral pain induced by this chemical irritant when used at this low dose. These latter results are in agreement with previous work showing that pain induced by similar doses of mustard oil was reversed by TRPA1 antagonism (Pereira et al., 2012).

When a higher dose of intracolonic mustard oil was used (2.5%) the results we obtained were diametrically different. RTX or AP18 induced some amelioration in nociceptive responses at the initial stage after the intracolonic administration of mustard oil, but as early as 5 min after the administration of the chemical irritant, the nociceptive responses were no longer modified by treatment with either RTX or AP18. Referred hyperalgesia after 2.5% mustard oil application also showed limited (nonsignificant) sensitivity to AP18, and no sensitivity to RTX. Therefore, during the first 5 min after application of this high dose of mustard oil, TRPA1 activation had some impact on the behavioral effects observed, whereas at later time-points (5–20 min) visceral pain appeared to become independent of this mechanism.

We observed that the intracolonic administration of the high dose of mustard oil induced marked histological alterations in the colon. These included prominent edema, which was absent after the ablation of TRPV1+ neurons. TRPA1 activation is known to result in the local release of neuropeptides such as substance P or calcitonin gene-related peptide (CGRP) among others, which produce vasodilatation and increased vascular permeability, thus promoting the appearance of edema (Engel et al., 2011; Matsuda et al., 2019). Therefore, in mice with TRPV1+ neuron ablation, the absence of edema after the high dose of mustard oil may be attributable to TRPA1 actions. However, since RTX did not affect either the late nociceptive responses or referred hyperalgesia despite the marked inhibition of mustard oil-induced edema, the participation of this neurogenic inflammation on visceral pain induced by 2.5% mustard oil appears to be limited.

Using pERK1/2 as a marker for neuronal activation and central sensitization after noxious stimulation (Gao and Ji, 2009), we found that 2.5% mustard oil induced substantial neuronal activation primarily in the superficial dorsal horn but also around the central canal (lamina X), in full agreement with previous studies that used equivalent markers for neuronal activation (*Fos* expression) (Traub and Murphy, 2002). Interestingly, we show that the ablation of TRPV1+ neurons eliminated only a limited portion of pERK1/2 + cells in the superficial dorsal horn, without affecting pERK1/2 in lamina X, which is fully consistent with the selective location of the central terminals of TRPV1 afferents in lamina I and outer lamina II (Cavanaugh et al., 2009). Therefore, a substantial part of neuronal activation after intracolonic administration of the high dose of mustard oil was independent of TRPV1 neurons (and hence unrelated to TRPA1 actions), which may explain why nociceptive behaviors and referred hyperalgesia may still be present despite ablation of the putative neuronal target of mustard oil. These results clearly point to a role of additional mechanisms different from TRPA1 activation in driving pain behaviors after the intracolonic administration of 2.5% mustard oil.

In addition to the edematous response in the colon induced by the high dose of mustard oil, we also observed severe damage to the colonic epithelium, which was independent of TRPV1-expressing neurons given that it was not modified by the ablation of TRPV1+ nociceptors with RTX. It has been reported that mustard oil is able to break the tight junction barrier, at least in cultured gastric epithelial cells, in a TRPA1-independent manner (Tashima et al., 2014), and this effect may partially explain the tissue damage we report here. Cytosolic factors released after cell death are known to constitute rapid nociceptive signals, and cytosolic ATP is one of the most critical messenger molecules in triggering nociception after this process (Cook and McCleskey, 2002). Sensory neurons express purinergic receptors of the P2X family for the detection of extracellular ATP, and we show that the P2X antagonist TNP-ATP was able to markedly decrease both late nociceptive responses and referred hyperalgesia induced by 2.5% mustard oil, which indicates that ATP (and not TRPA1 activation) contributes to pain behavior. To our knowledge, this is the first report showing that the effects induced by mustard oil on pain behavior can be reversed by a purinergic antagonist. However, TNP-ATP has been reported to decrease nociceptive responses or neuronal activation induced by visceral stimulation with acetic acid (Honore et al., 2002; Xu et al., 2008), which–like a high dose of mustard oil–may be able to induce tissue damage with the consequent ATP release. It is worth noting that TNP-ATP is a competitive antagonist with nanomolar affinity at P2X1 and P2X3 receptors, whereas it is several orders of magnitude less potent at other P2X receptors (reviewed by North and Jarvis, 2013). P2X1 receptors are expressed in the DRG at negligible levels (Xu and Huang, 2002; Chen et al., 2016), but are highly expressed in smooth muscle and platelets (Mahaut Smith et al., 2019). Therefore, it is unlikely that P2X1 receptors are involved in the nociceptive effects induced by 2.5% mustard oil. However, the P2X3 subtype has been well studied in pain physiology (Burnstock, 2016). It is known that peptidergic and nonpeptidergic nociceptors are well segregated neuronal populations in the mouse (Zwick et al., 2002; Price and Flores, 2007; Montilla-García et al., 2018), and while P2X3 receptors are expressed by nonpeptidergic neurons in this species, TRPV1 is expressed by peptidergic nociceptors (Zwick et al., 2002). Therefore, taking into account that ATP is sensed by P2X-expressing neurons which differ from TRPV1-expressing nociceptors, P2X activation by ATP released after tissue injury may partly explain both pERK activation in the spinal cord and the finding that pain behaviors in response to a high dose of mustard oil are apparently independent of TRPV1+ neurons, as reported here.

Scientific evidence for the role of TRPA1 and P2X3 receptors in pain processing has led to the development of selective antagonists for both targets. Several TRPA1 antagonists are being tested in clinical trials for postoperative or neuropathic pain treatment, and are currently receiving clinical scrutiny (Souza Monteiro de Araujo et al., 2020). Thus far, none of them have been reported to induce side effects, but definitive data on their efficacy and safety are not yet publicly available (Souza Monteiro de Araujo et al., 2020). In addition, P2X3 antagonists are undergoing clinical trials, with promising results for osteoarthritic pain and with a good safety profile, since only taste disturbances (hypogeusia or dysgeusia) have been reported to be frequent after treatment (Krajewski, 2020). Although both TRPA1 and P2X have been suggested as promising pharmacological targets to treat visceral pain (e.g., Lapointe and Altier, 2011; Burnstock, 2017), our findings show that when visceral pain is caused by tissue injury, pain relief by P2X antagonism appears to be much more effective than TRPA1 inhibition. Patients with inflammatory bowel diseases such as Crohn’s disease and ulcerative colitis show macroscopic painful lesions in the colon (Gecse and Vermeire, 2018). Therefore, we can speculate that purinergic antagonism may be more likely to show efficacy than TRPA1 inhibition in this patient population.

It is worth noting that TNP-ATP did not fully reverse pain behaviors or referred hyperalgesia induced by the high dose of mustard oil, suggesting that other mechanisms are involved in pain behavior apart from TNP-ATP-sensitive P2X receptors. These might include other purinergic receptors (insensitive to TNP-ATP), as well as a myriad of additional pronociceptive mediators such as glutamate, kinins, cytokines and trophic factors, which–like ATP–are known to contribute to pain after tissue injury (Amaya et al., 2013). Therefore, our finding of a partial effect of TNP-ATP on visceral pain after 2.5% mustard oil-induced tissue injury may be explained by the simultaneous participation of additional mechanisms complementary to P2X activation.

**FIGURE 9:**
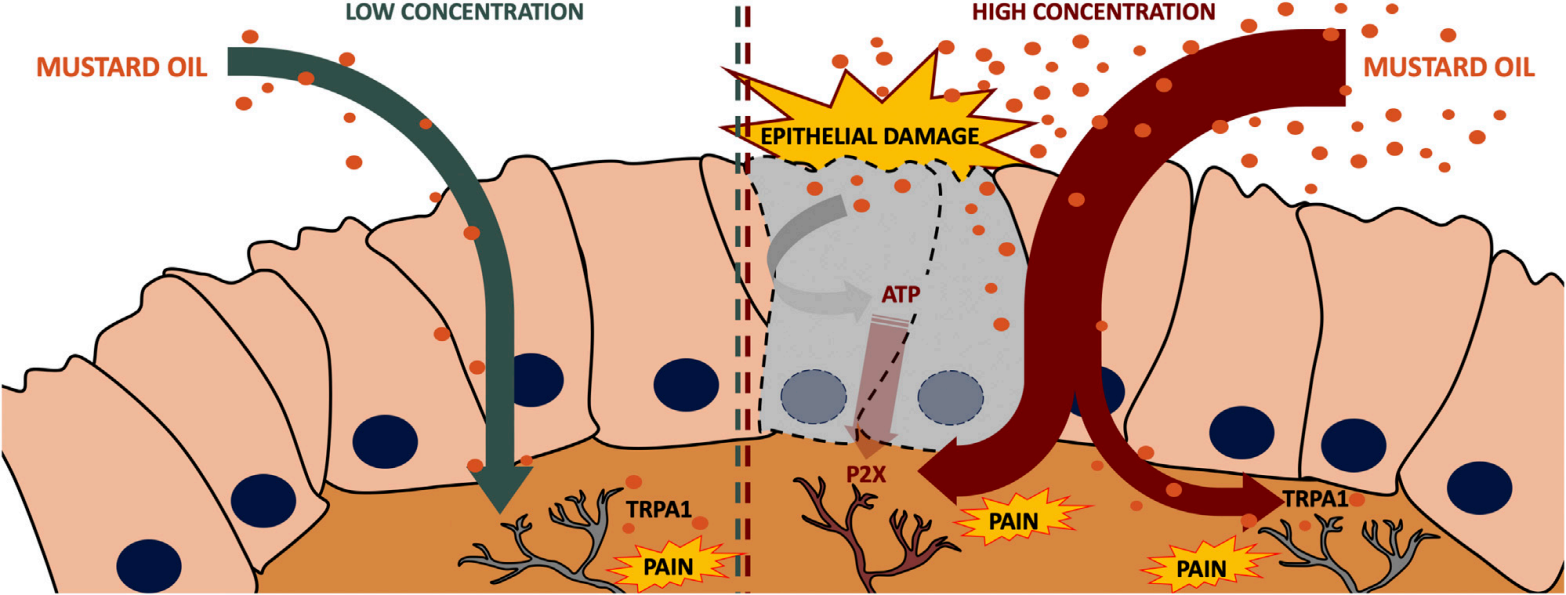
Proposed mechanisms of the effects induced by a low and a high dose of mustard oil. A low dose (0.5%) of intracolonic mustard oil induces visceral pain in a manner fully dependent on TRPA1 actions, whereas when a high dose (2.5%) of this chemical irritant is used, visceral pain is heavily influenced by P2X receptors putatively activated by ATP released by the damaged tissue.

In conclusion, we found that a low dose (0.5%) of intracolonic mustard oil induces visceral pain in mice, in a manner fully dependent on TRPA1 actions, whereas when a high dose (2.5%) of this chemical irritant is used, visceral pain becomes independent of TRPA1 activation as soon as 5 min after administration, and is influenced by P2X receptors purportedly activated by ATP released in the damaged tissue. The proposed mechanisms for the effects of a low and high dose of mustard oil are illustrated in Figure 9. Therefore, TRPA1 inhibition is not sufficient to substantially decrease visceral pain resulting from tissue injury, whereas purinergic antagonism appears to be a more effective strategy.

## Data Availability Statement

The raw data supporting the conclusions of this article will be made available by the authors, without undue reservation.

## Ethics Statement

Animal care was in accordance with institutional (Research Ethics Committee of the University of Granada, Spain), regional (Junta de Andalucía, Spain) and international standards (European Communities Council Directive 2010/63).

## Author Contributions

Conceptualization, RG-C, MC, JMB and EJC; Data Acquisition, RG-C, AM-G, GP, JMT, JC and EF-S; Writing – Original Draft: RG-C, JMB and EJC; Writing – Review & Editing, all authors; Funding Acquisition, JMB and EJC.

## Funding

This study was partially supported by the Spanish State Research Agency (10.13039/501100011033) under the auspices of MINECO (grant number PID2019-108691RB-I00), and by the Junta de Andalucía (grant CTS-109).

## Conflict of Interest

The authors declare that the research was conducted in the absence of any commercial or financial relationships that could be construed as a potential conflict of interest.
