# Supplementary material for: Intracolonic Mustard Oil Induces Visceral Pain in Mice by TRPA1-Dependent and -Independent Mechanisms: Role of Tissue Injury and P2X Receptors
Source: Front Pharmacol. 2021 Jan 21;11:613068. doi: 10.3389/fphar.2020.613068 (PMC7859884; doi:10.3389/fphar.2020.613068)
Supplement: Supplementary file 1 [file image1.pdf]

Figure S1

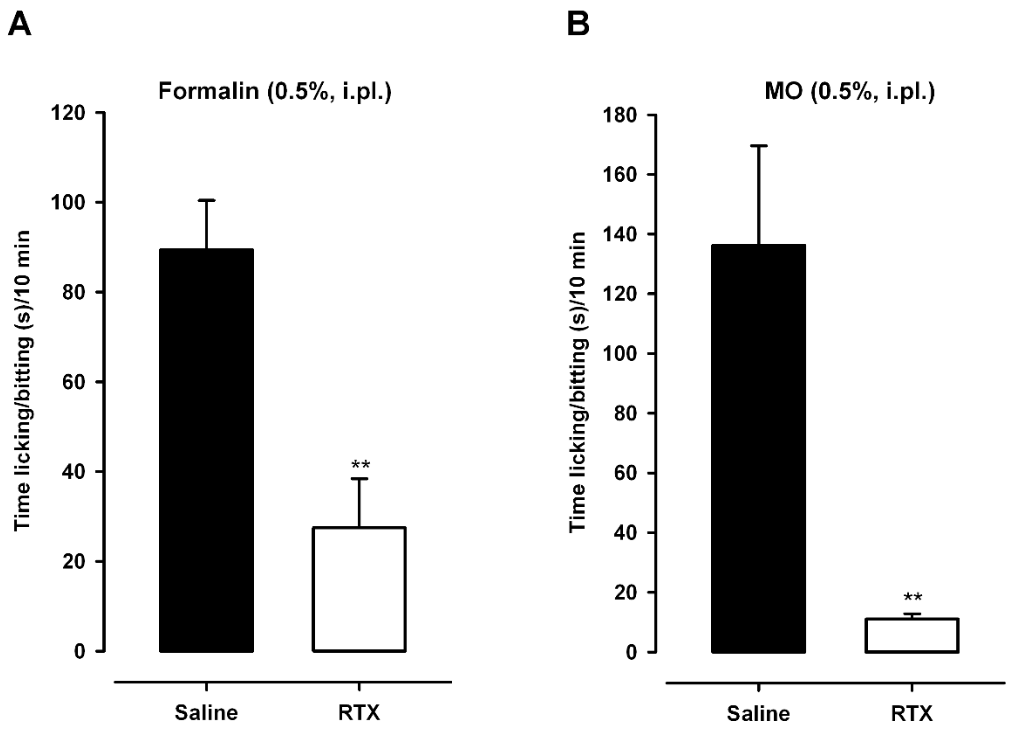

Figure S1. Ablation of TRPV1-expressing neurons markedly reduces nociceptive responses to intraplantar formalin or mustard oil. Nociceptive responses (duration of licking or biting the paw) in mice treated with resiniferatoxin (RTX) or its solvent (saline), during the 10 min immediately after the intraplantar administration of (A) 0.5% formalin or (B) 0.5% mustard oil (MO). Each bar and vertical line represents the mean ± SEM of values obtained in 6–8 mice per group. Statistically significant differences between the values in mice treated with RTX or saline: $^{**}p < 0.01$ (Student's *t* test).
